# Community-based rehabilitation service in Chengdu, Southwest China: a cross-sectional general survey

**DOI:** 10.1186/s12913-020-05480-3

**Published:** 2020-07-08

**Authors:** Siliang Chen, Yi Lei, Hua Dai, Jia Wu, Ziyu Yang, Xiaoyang Liao

**Affiliations:** 1 West China School of Medicine, Sichuan University, Chengdu, 610041 Sichuan PR China; 2 International medical center/center of general practice, West China Hospital, Sichuan University, Chengdu, 610041 Sichuan PR China

**Keywords:** Community-based rehabilitation, General survey, Primary health center

## Abstract

**Background:**

World Health Organization initiated community-based rehabilitation (CBR) in 1978, and by now, it has been an essential process of medical services worldwide. China had strengthened primary health care on building more than 35,000 community health centers (CHCs) in cities, and more than 34,000 township health centers (THCs) in the rural area. Nevertheless, it remains unclear that if these primary health centers could provide optional rehabilitation services for disabilities. And this study aims at evaluating the supply capacity of rehabilitation service in primary health centers of Chengdu, a regional center city of southwest China.

**Method:**

We conducted a general investigation of primary health centers in Chengdu, a city located in southwest China with more than 15 million population. Our investigation covered all of Chengdu’s 390 primary health centers from October to November 2016. We researched these primary health centers on basic rehabilitation services, diseases, and rehabilitation equipment quantity and quality, and traditional Chinese medicine (TCM) physiotherapy.

**Result:**

Rehabilitation therapy is available in 88.9% (337 of 379) of all primary health centers. Meanwhile, CHCs slightly surpass THCs with an available rate of 92.2% (106 of 115) and 87.5% (231 of 264), respectively. Traditional Chinese Medicine (TCM) physiotherapy is available in 97.1% (368 of 379) of all primary health centers, 97.3% (112 of 115) of CHCs and 97.0% (256 of 264) of THCs. Quantitative analysis showed that substantial factors which could make an impact on the number of patients per year contain: categories of rehabilitation disease (*P* < 0.001, 95% confidence interval (CI) [− 1.571, − 0.702]),number of rehabilitation bed (P < 0.001, 95%CI [− 1.249, − 0.290]).

**Conclusion:**

CBR and TCM physiotherapy has become accessible for disabilities in most basic health centers of Chengdu City, whereas, available rate of CBR in THCs is lesser than in CHCs, which suggests an imbalance in primary health service development between rural and urban area. Categories of rehabilitation diseases, and the number of rehabilitation beds constitute co-factors that make an impact on the CBR capacity of basic health centers.

## Background

Community-based rehabilitation (CBR) has been a widely-accepted pattern for individuals to get access to convenient, flexible and economic rehabilitation services since its implementation in the 1970s [1]. A strategy called community-based rehabilitation was launched by the World Health Organization in 1978 to meet the need of rehabilitation services around the world [2]. Since being initiated, CBR has been introduced into more than 90 countries as a rational pattern to deliver rehabilitation services. Statistics show that more than 1 billion people are with disabilities globally. And about 110–190 million adults and 93 million children are experiencing significant difficulties in daily function [3]. The World Health Organization (WHO) set CBR as a critical development direction for disabilities, according to the *Global Disability Action Plan* [3]*.*

After being introduced into China in the 1980s, rehabilitation services were mainly provided by volunteers in the early stage. However, due to the lack of systematic training for volunteers, CBR service remains in a low quality for many years. With more investment and support from the government, rehabilitation services based on communities began to develop in a rapid speed to alleviate the lack of rehabilitation services for disabilities [4]. Nevertheless, inadequate financial support and lack of rehabilitation physicians have hindered development of CBR services [5].

There are 34,522 CHCs and 36,871 THCs in mainland China by Sep, 2015 [6]. Even though the Chinese government has devoted significant resources in building primary health centers in recent years, rehabilitation services and personnel are still largely in need. In the next 10 years, it is estimated that China required 60,000 rehabilitation physicians, 150,000 rehabilitation therapists and 60,000 nurses to meet the demand [7]. By the end of 2010, China had more than 85 million disabled persons, according to the census [8]. But until 2018, there were only 9036 professional rehabilitation institutions and about 250,000 staff in these institutions, including physicians, nurses and administrative [9], which indicated that most of the disabled persons in mainland China could not get easy access to rehabilitation service. On the patients’ side, they prefer to go to hospitals rather than primary health care centers for what they believe in having more professional service [10]. Consequently, the Chinese government initiated the “Healthy China 2030” plan, in which service capacity and scale of primary health care system was set as a focused point to invest [11].

Nevertheless, there are rare data about how widely the primary health centers could cover disabilities who require convenient and economic rehabilitation services. Because there are many challenges during the process of CBR evaluation would hamper investigators to get reliable data. For instance, community health centers in the vast rural area of China are difficult to be evaluated because of the low literacy and the large variety of dialects. In addition, participants tend to select the midpoint and skip some items because Chinese culture affects their behavior and drives them prefer to be modest and to underestimate their performance [12–14]. They are ashamed to openly discuss and rate their family or organization [15]. Hence, we designed and performed this cross-sectional survey to evaluate the supply capacity of rehabilitation services in primary health centers of Chengdu and find out how we could promote the development of community-based rehabilitation. We purposely conducted this survey among the leaders of primary health centers rather than patients, which may get objective data and minimize bias caused by Chinese patients’ inclination to rate their performance and refrain from exaggerating their achievements modestly.

## Methods

### Subjects

To effectively evaluate CBR in CHCs, we selected Chengdu, the capital of Sichuan province, in southwest China with a land area of 12,390km^2^ and a population of 16.33 million by 2018 [16, 17]. This city is the economic and cultural center of western China, and about 34.49% of the population dwell in rural areas.

And we conducted a general cross-sectional survey of all 390 primary health centers in Chengdu. To reduce the uncertainty caused by the large variety of education levels of patients, the survey questionnaire was undertaken by lead physicians in these community health facilities from October 2016 to November 2016. At the same time, data about these community health facilities was obtained from the Health Commission of the Sichuan Province to verify survey results. In this way, we expected to receive as accurate and objective data as we could.

In summary, there are two critical directions for China to enhance CBR: the first is to improve the scale of CBR service in primary health centers; the second is to improve service quality to attract patients instead of sending them to superior hospitals. To evaluate priamry health centers from these two aspects, we selected data of the number of patients (person-time) in 2015 as a proxy of CBR service capacity. Since this number could directly reflect the size and volume of CBR service. On the other hand, the larger this number was, the more patients the primary health center attracted, which indicated the higher quality of CBR service.

### Development of the questionnaire

The questionnaire was designed according to the policy of <Service quality evaluation guideline for primary care facilities> [18], made by the National health and family planning commission of the people’s republic of China in 2016, and modified after consideration of applicability as well as expert judgment. We focused on evaluating the basic capabilities of primary care facilities in providing services Thus we mainly collected the following variables:

1. Total number of rehabilitation patients of each primary health facilities in 2015
2. Total number of disabilities in the area under the jurisdiction of each primary health facilities in 2015
3. .Categories of rehabilitation diseases
4. Basic conditions: served population, area of the structure, the total number of rehabilitation physicians, physical therapy equipment, cervical and lumbar traction equipment, infrared therapy apparatus, ultrasound therapy apparatus, number of rehabilitation bed, number of therapeutic rehabilitation room.
5. Rehabilitation training: rehabilitation lecture, rehabilitation counseling.
6. Rehabilitation management measures: rehabilitation training plan, rehabilitation-related system, self-inspection.

The questionnaire is available in additional file 1.

### Administration of the questionnaire

An instruction for data quality control was developed for the questionnaire and then distributed by local health bureaus to the leaders of each primary health center. Three hundred ninety lead physicians completed the questionnaires independently and submitted them to the survey team in 2 weeks after receiving them. Then the survey team staff rechecked the collected questionnaires and filled the missing data by interviewing leaders of primary health centers through telephone. At last, the team randomly sent staff to 5% of the CHC facilities to validate data received.

### Data analysis

Up to now, qualitative methodologies have dominated the field of evaluations in community-based rehabilitation, but quantitative methods have demonstrated strong potential in capture a better assessment [19]. Therefore, quantitative data analysis was adopted in our cross-sectional survey. Data were logged into Epidata by dual investigators and analyzed with SPSS 22.0 (SPSS Inc., Chicago, IL, USA).

Multivariable ordinal logistic regression analysis was used to determine which specific characteristics were independently related to the number of patients per year in CHCs and THCs—among the number of diseases, rehabilitation diseases, area of the structure, rehabilitation equipment, rehabilitation management measures, etc. All significant tests were 2-tailed, and those with a *P*-value < 0.05 were considered statistically significant. In addition, we also conducted descriptive analysis on the basic condition of CBR service in primary health centers.

## Results

Of all the 390 CHCs this survey covered, 379 primary health centers responded to this survey, which came out with a response rate of 97.2%. After data validation, all 379 feedbacks entered into the analysis.

The first part is the basic status of community rehabilitation and TCM physiotherapy (Table 1.). Rehabilitation service is available in 88.9% (337 of 379) of all primary health centers, meanwhile, CHCs slightly surpass THCs with an available rate of 92.2% (106 of 115) and 87.5% (231 of 264), respectively. Traditional Chinese Medicine (TCM) physiotherapy is available in 97.1% (368 of 379) of all primary health centers, 97.3% (112 of 115) of CHCs and 97.0% (256 of 264) of THCs. And the top 5 diseases of community rehabilitation in descending order are the cervical vertebral disease, lumbar vertebral disease, osteoarthritis, adhesive capsulitis, and stroke sequelae.

**Table 1:** The status of community rehabilitation and TCM physiotherapy

	urban community(*N* = 115)		rural community (*N* = 264)	
	yes	no	yes	no
Rehabilitation	106	9	231	33
TCM physiotherapy	112	3	256	8
both	106	9	231	33

Statistics about the basic condition of CHCs read that the total number of rehabilitation patients in 2015 were more than 2 million person-time. The average number of patients of CHCs and THCs were 7456.9 and 4762.6 person-time in 2015, respectively. (Table 2.)

**Table 2:** The basic condition of primary health centers

	primary health centers				
	Total of CHCs (*N* = 115)	Average of CHCs	Total of THCs (*N* = 264)	Average of THCs	Total (*N* = 379)
Number of patients^a^(person-time)(KF01)	857544^e^	7456.9	1,257,323	4762.6	2,114,867
Number of disabilities^b^(KF05)	69,037	600.3	156,438	592.6	225,475
Number of disabilities with health record^c^(KF06)	47,007	408.8	109,166	413.5	156,173
**Basic condition**					
Building area^d^(RY02)(square meter)	376,090.85	3270.4	893,922.37	3386.1	1,270,013.22
Number of rehabilitation physicians (RY08)	215	1.9	323	1.2	538
**Rehabilitation equipment**					
Cervical and lumbar traction equipment (KF09)	107	0.9	243	0.9	350
Infra-red ray therapy apparatus	93	0.8	188	0.7	281
Ultrasound therapy equipment	60	0.5	93	0.4	153
Rehabilitation beds (KF02)	1274	11.1	2270	8.6	3544
Number of rehabilitation therapeutic room	290	2.5	495	1.9	785
**Rehabilitation training**					
Rehabilitation lecture^f^(times)(KF07)	847	7.4	1381	5.2	2228
Rehabilitation counseling^f^ (times)(KF08)	21,748	189.1	9914	37.6	31,662
**Rehabilitation management measures**					
rehabilitation training plan (number of launch)^g^ (KF12)	95	0.8	189	0.7	284
Rehabilitation related system (number of launch) (KF13)	104	0.9	205	0.8	309
Self-inspection (number of launch) (KF14)	86	0.7	179	0.7	265

^a^.Number of patients: number of all patients received CBR service in 2015, counted in person-time

^b^.Number of disabilities: total number of disabilities in the service area

^c^.Number of disabilities with health record: total number of disabilities with health documents in primary health centers and visit for CBR regularly in the service area

^d^.Building area: total building area of primary health centers

^e^.This number indicates the total number of patients in all 115 CHCs

^f^.Rehabilitation lecture and counseling in 2015

^g^.Number of launch: total number of primary health centers launching this measure

To quantitively analyze potential factors that could influence the capacity of CHCs rehabilitation service, we covert the dependent variable, the number of rehabilitation patients to level variable according to quartile. (Table 3.)

**Table 3:** Conversion of the number of rehabilitation patients

Variable	Classification criteria
Number of patients (person-time/year)	1:< 378; 2:> = 378 AND < 2375; 3:> = 2375 AND < 7255; 4:> = 7255

Quantitively analysis indicated that substantial factors which could make an impact on the number of patients per year contain: species of rehabilitation disease (*P* < 0.001, 95% confidence interval (CI) [− 1.571, − 0.702]).number of rehabilitation bed (*P* < 0.001, 95%CI [− 1.249,-0.290]) (Table 4.)

**Table 4:** Multivariable ordinary logistic regression analysis on the number of patients of primary health centers in Chengdu

		β	Wald	P	95% Confidence Interval	
					Lower limit	Higher limit
Number of disabilities (KF05)	< 248	− 0.032	0.011	0.92	− 0.630	0.565
	> = 248 AND < 486	0.174	0.300	0.58	−0.447	0.794
	> = 486 AND < 840	0.144	0.219	0.64	−0.460	0.748
	> = 840	reference				
Rehabilitation diseases (KF03)	< 9	−1.136	26.242	0.00	−1.571	− 0.702
	> = 9	reference				
Served population^a^ (RY01)(10 thousand)	< 1.8	−0.561	2.827	0.09	−1.216	0.093
	> = 1.8 AND < 3	−0.628	4.646	0.03	−1.198	− 0.057
	> = 3 AND < 5.94	−0.194	0.468	0.49	−0.750	0.362
	> = 5.94	reference				
Building structure (RY02)(square meter)	< 1600	−0.687	3.816	0.05	−1.376	0.002
	> = 1600 AND < 2628.4	0.157	0.276	0.60	−0.428	0.741
	> = 2628.4 AND 4296	−0.297	1.091	0.30	−0.855	0.260
	> = 4296	reference				
Number of rehabilitation physicians (RY08)	<=1	−0.438	3.761	0.05	−0.882	0.005
	> 1	reference				
Cervical and lumbar traction equipment (KF09)	Yes	−1.047	1.352	0.25	−2.811	0.718
	No	reference				
Infra-red ray therapy apparatus	No	0.016	0.004	0.95	−0.507	0.540
	Yes	reference				
ultrasound therapy equipment (KF11)	No	−0.196	0.758	0.38	−0.638	0.245
	Yes	reference				
Rehabilitation bed	<=7	−0.770	9.908	0.00	−1.249	− 0.290
(KF02)	> 7	reference				
Number of rehabilitation therapeutic room (KS0301)	<=2	−0.148	0.389	0.53	−0.614	0.317
	> 2	reference				
rehabilitation training plan (KF12)	No	−0.289	0.848	0.36	−0.905	0.326
	Yes	reference				
Rehabilitation self-examination (KF14)	No	0.390	1.943	0.16	−0.158	0.939
	Yes	reference				

^a^Served population: number of all inhabitants in the service area

### Discussion

To authors’ knowledge, by Sep 2019, this survey is the ever first general survey of rehabilitation capacity of primary health care in China from a literature study, as well as the first analysis of factors that influence rehabilitation service capacity in the primary health system. In the general survey, we found more than 88% of CHCs and THCs involved in this survey have been well equipped to provide rehabilitation services. But, the imbalance between urban and rural community health centers remains prominent, even though the government has made efforts to minimize the gap. For nearly the same average number of disabilities (600.3 of CHCs, 592.6 of THCs), CHCs’ number of patients is 1.56 times as THCs’ (7456.9 vs 4762.6). Compared to THCs, CHCs obviously equipped with higher-quality equipment, more rehabilitation doctors (1.9 vs 1.2) and more rehabilitation counseling (189.1 vs 37.6). Reasons that make this kind of gap between urban and rural may be various, such as economic level, physician attraction, transportation facilities, and citizens’ health awareness. Consequently, this phenomenon of the urban-rural imbalance inspires the government and community to shift more financial support and health education to the rural area to improve the rehabilitation capacity of primary health centers there.

As to factors that were analyzed by multivariable ordinary logistic regression, categories of rehabilitation diseases and the number of rehabilitation bed are considered statistically significant. However, we should not ignore the number of rehabilitation physicians as a potential factor even though it is not statistically significant (*P* = 0.05, 95%CI [− 0.882,0.005]). For instance, β value of categories of rehabilitation diseases is − 1.136, which means primary health centers house more than 9 categories of rehabilitation diseases would attract a larger number of patients, comparing to those with less than 9. It indicates that increase the types of rehabilitation diseases would improve CBR service capacity, which should be considered during the planning of the primary health care system. Following the same logic, increasing the number of rehabilitation beds and physicians should also be included in the plan when possible.

Since 2009, the Chinese government has been putting a large number of resources to improve its primary health care system. For example, in 2014, the expenditure on primary care reached ¥110 billion [20]. In less than 10 years, the infrastructures and facilities of the CHCs and THCs were significantly enhanced [21].

Along with the giant leap in the primary health care system under a powerful and effective push from China government on medical services, the rehabilitation capacity of these primary health centers also advanced in both hardware and software. However, in the southwest of China, there are dozens of regions troubling in short of appropriate medical service, and inevitable lack of affordable and convenient rehabilitation services, because of poverty, inadequate transportation and lack of health awareness. Chengdu, as the central city of southwest China, has a powerful and comprehensive influence on regional development in every aspect, including economy, education, culture, technology, and medicine. The outcome of our study, therefor, may be representative to entire southwest China rather than a city.

Due to poor education and training systems on general practitioners, rehabilitation services could not be well delivered to disabilities, even though CHCs have been equipped with the newest equipment and technology. Hence, creating an optimal general practitioner training system would be another driver that could further improve rehabilitation service capacity in the primary health system.

## Limitation

In this survey covering all 390 basic primary health centers of Chengdu city, we eventually received 379 intact replies that could be used in analysis. Other 11 primary centers were ruled out from analysis due to incomplete data, which may lead to unknown bias in the results. Another limitation is that we selected the number of patients as a proxy of CBR service, which we think could reflect the scale and service quality of a primary health center. Patients did not know exactly how a primary health center is, they would select which is better according to their experience and impression during accepting CBR service. Hence, patients’ subjective judgement decides whether to accept CBR service in CHCs and then has an influence on our results.

## Conclusion

Rehabilitation service capacity of primary health facilities in Chengdu took a giant leap in the past 20 years and reached a relatively high level in both quantity and quality. However, there are still many flaws and shorts during the rapid development of the primary health system and rehabilitation service. For instance, the imbalance between CHCs and THCs is one of the challenges that need to be addressed. Our study shows that the government could take measures directly correlated with rehabilitation service capacity to improve CBR service capacity, such as house more categories of rehabilitation diseases in the center, train more rehabilitation physicians and augment the number of rehabilitation beds.

## Additional Files

**Additional File 1.** Self-evaluation form of basic medical service capacity of primary medical institutions in Chengdu

## Data Availability

The datasets used and analyzed during the current study are available from the corresponding author on reasonable request.

## Abbreviations

CBR: Community-based rehabilitation
CHC: Community health center
THC: Town health center
TCM: Traditional Chinese medicine
CI: Confidence interval
