# Supplementary material for: Community-based rehabilitation service in Chengdu, Southwest China: a cross-sectional general survey
Source: BMC Health Serv Res. 2020 Jul 8;20:625. doi: 10.1186/s12913-020-05480-3 (PMC7346348; doi:10.1186/s12913-020-05480-3)
Supplement: Supplementary file 1 — Additional File 1. Self-evaluation form of basic medical service capacity of primary medical institutions in Chengdu [file 12913_2020_5480_MOESM1_ESM.docx]

**Self-evaluation form of basic medical service capacity of primary medical institutions in Chengdu**

**Precautions**

1. This self-assessment form needs to fill in paper documents and online electronic documents, and the two must be filled in the same. It is recommended that you fill in paper documents first, and then fill in the electronic documents after repeated confirmation. The electronic documents cannot be modified once submitted.
2. The notes for filling in the self-assessment form paper documents are as follows:

2.1 The self-assessment form should be filled with a "black" signature pen, and each item must be filled in according to the "remarks" requirement. No items can be omitted or left blank.

2.2 Please mark an X in a checkbox in each row to answer each question, as shown in the figure: ☒ . Specify the number in the provided horizontal line, as shown in the figure:   5  .

2.3 If the paper document of the self-assessment form is filled in incorrectly, keep the original handwriting, draw a small horizontal line on it, fill in the correct data next to it, and sign the name and date of the modifier next to it. No mistakes should be made with correction fluid, knife or black. As shown: ~~100~~   111 Zhang San 2016.9.1.

1. In the self-assessment form, the unit ID must be 12 digits or 13 digits, and the district (county) ID must be 3 digits. Please refer to Annex 3 . Such as: A District B Road Community Health Center ID: 510 000 004 062 , district (county) ID: 101 .
2. If you have any questions, please send them to nlpg2016@126.com.

1

Institution name:

Unit ID (refer to Annex 3 ):

District (County):

District (County) ID (refer to Annex 3) :_______________________

Unit Nature: □ 1. Community Health Service Center          □ 2. Township Health Center

work phone:

Person in charge of medical institution: Person in charge:_________________ Phone:

Filler:_______________

Date of filling:

Filler Phone:

Reviewer (paper document):

Reviewer's Phone (paper document):

Reviewer (Internet electronic document):

Reviewer's Phone (Internet electronic document):

**Condition of service**

**(A) (RY) Staffing**

1. (RY01) Serving population in the jurisdiction □□□ . □□ thousand.

   (Note: The service population refers to all permanent residents in the area served by the primary medical institution. Please fill in two decimal places. If not, please fill in “0”)

1. (RY02) Construction Area □□□□□□ . □□ square meters. (Note: the actual building area of ​​the medical institution)
2. (RY03) The unit covers an area of □□□ . □□ square meters. (Note: the actual area of ​​the medical institution)
3. (RY04) The number of the staff in the hospital .

   (Note: All serving staff in the hospital, including doctors, nurses, medical technicians, administrative staff, logistics staff and workers)

1. (RY05) the number of clinical practitioner in the hospital .
2. (RY08) The number of a physician engaged in rehabilitation .

**(C) (KF) Rehabilitation Service** (Whole Hospital or Center from 2015.01.01 to 2015.12.31)

1. (KF01) The number of patients per year_______ of rehabilitation diagnosis and treatment includes traditional Chinese medicine acupuncture, physiotherapy, traditional Chinese medicine

2. (KF02) The number of rehabilitation beds .

3. (KF03) The number of rehabilitation disease species / year.

4. (KF04) First five kinds of rehabilitation diseases :( first diagnosis, in descending order to fill in)

(1)

(2)

(3)

(4)

(5)

5. (KF05) the number of jurisdiction disabled people

6. (KF06) the number of archived disabled people in jurisdiction___

7. (KF07) whether held rehabilitation lectures or health consultations /times per year

8. (KF08) the number of Rehabilitation-related health consultations times/ year

9. (KF09) whether there is a cervical and lumbar traction device   □ 1 No       □ 2 Yes

10. (KF10) whether there is an infrared therapy machine                   □ 1 No       □ 2 Yes

11. (KF11) whether there is an ultrasonic treatment machine                 □ 1 No       □ 2 Yes

12. (KF12) whether there is a clear diagnosis for each patient recovery and rehabilitation program       □ 1 No       □ 2 Yes

13. (KF13) whether there is formulation of rehabilitation work rules            □ 1 No       □ 2 Yes

14. (KF14) whether the department have self-examination, evaluation and improvement measures for rehabilitation services         □ 1 No       □ 2 Yes
